# Atrial fibrillation in intensive care

**DOI:** 10.1186/2197-425X-3-S1-A209

**Published:** 2015-10-01

**Authors:** S Ganguly, T Brown, C Pritchett, S Edie, P Allan, M Spivey

**Affiliations:** Royal Cornwall Hospital, Critical Care, Truro, United Kingdom

## Intr

Atrial fibrillation (AF) is a commonly encountered complication in intensive care. The causes of AF are widespread & thought to be an electrolyte disturbance or altered intravascular volume, but recent evidence suggesting that vasopressor dose & raised MAP also are indicated. There is little uniformity or guidance in the management of this arrhythmia.

## Objectives

New onset atrial fibrillation occurs in about 10% of patients admitted to intensive care and has an adverse effect on several outcome measures. The objective of this audit was to assess the incidence of new onset AF in critical illness, its causes, treatments & to evaluate a proposed target as laid by the Royal College of Anaesthetists (RCOA).

## Methods

Using the Philips Intel iView Clinical Information Portfolio (ICIP), all patients who developed AF during their admission to ICU were identified between March 2012 & June 2014. The records were examined for the demographics, MAP,vasopressor dose, electrolytes immediately prior to development of AF. The drug charts were examined for evidence of rate & rhythm control & anticoagulation. In addition the use of DCCV & echocardiography were recorded.

## Results

During the time period there were 1900 admissions to the Unit. On examining the patient record, 203 patients developed AF during their ICU admission. In 60% patients the MAP was ≥75 mmHg, with 7% on a dose of noradrenaline > 0.3 mcg/kg/min. Nearly half of the patients had a diagnosis of severe sepsis or septic shock & in this group the MAP≥75 mmHg in 60%.31% patients had rhythm control, 14% rate control & 7% both. Anticoagulation was prescribed in only 15% patients. 38 patients had a documented ECHO. The median values for K^+^ & Mg^2+^ at the onset of AF were 4.3 & 0.85 respectively.

## Conclusions

The incidence of new AF in our ICU was 11%. It was not just confined to those with greater disease severity as defined by APACHE, but seen predominantly in those with APACHE II < 25.Interestingly the relation between higher MAP& AF seen in the SEPSISSPAM was repeated in our patient cohort. As a common practice ensuring K^+^ & Mg^2+^ greater than 4.5 mmol/l &1 mmol/l respectively to treat AF seems to be an appropriate management strategy in absence of well documented evidence. Small proportion of patients received an ECHO, which we believe to be a result of the constraints on the echocardiography service. We know that transthoracic echocardiography (TTE) diagnosed left ventricular dysfunction, increased left atrial diameter and valvular abnormality were associated with increased risk of stroke, thromboembolism and mortality. Hence it's both clinically relevant & cost effective to perform an ECHO. Lastly few patients received the standard of care documented by the RCOA.
